# Tumor spheroids under perfusion within a 3D microfluidic platform reveal critical roles of cell-cell adhesion in tumor invasion

**DOI:** 10.1038/s41598-020-66528-2

**Published:** 2020-06-15

**Authors:** Yu Ling Huang, Yujie Ma, Cindy Wu, Carina Shiau, Jeffrey E. Segall, Mingming Wu

**Affiliations:** 1 Department of Biological and Environmental Engineering, Cornell University, Ithaca, NY 14853 USA; 2 Anatomy and Structural Biology, Albert Einstein College of Medicine, 1300 Morris Park Avenue, Bronx, 10461 New York USA

**Keywords:** Cancer microenvironment, Cancer models, Cell adhesion, Cell migration, Motility, Biological models, Biophysical methods, Lab-on-a-chip, Biological techniques, Biophysics, Cancer, Cell biology

## Abstract

Tumor invasion within the interstitial space is critically regulated by the force balance between cell-extracellular matrix (ECM) and cell-cell interactions. Interstitial flows (IFs) are present in both healthy and diseased tissues. However, the roles of IFs in modulating cell force balance and subsequently tumor invasion are understudied. In this article, we develop a microfluidic model in which tumor spheroids are embedded within 3D collagen matrices with well-defined IFs. Using co-cultured tumor spheroids (1:1 mixture of metastatic and non-tumorigenic epithelial cells), we show that IFs downregulate the cell-cell adhesion molecule E-cadherin on non-tumorigenic cells and promote tumor invasion. Our microfluidic model advances current tumor invasion assays towards a more physiologically realistic model using tumor spheroids instead of single cells under perfusion. We identify a novel mechanism by which IFs can promote tumor invasion through an influence on cell-cell adhesion within the tumor and highlight the importance of biophysical parameters in regulating tumor invasion.

## Introduction

The human body is composed mostly of fluids (~60% the body mass), and approximately one third of the body fluid is in constant movement through the blood circulation as well as the interstitial extracellular space^1^. The movement of the fluids within the interstitial space, or interstitial flows (IFs), is driven by the hydrostatic and osmotic pressure differences among the blood, venous and lymphatic vessels^2^. IFs are critical for maintaining healthy tissue homeostasis and are known to provide necessary nutrients and at the same time remove metabolic wastes from tissues^1,3^. In healthy tissue, interstitial flow rates were reported to be less than 2.0 µm/s with an average of 0.6 µm/s using a rabbit ear model^4^. In the tumor microenvironment, IFs are often elevated due to high interstitial fluid pressure within the tumor as a result of abnormal angiogenesis, and were reported to be up to 9 µm/s in animal models^5–7^ and up to 55 µm/s in human patients^5^. Although the importance of the tumor microenvironment in tumor progression has been emphasized in recent literature, studies on roles of IFs within the microenvironment in tumor invasion are still at an early stage.

IFs can impact tumor invasion via redistribution of signaling molecules within the tumor microenvironment and/or direct application of external stresses onto the cells^8–11^. Using a modified 3D Boyden Chamber assay, pioneering work from the Swartz lab showed that IFs can facilitate tumor secreted chemokine gradients along the direction of the flow and subsequently guide tumor cell migration along the flow direction via chemotaxis, a phenomena known as autologous chemotaxis^12,13^. Using a 3D microfluidic platform, the Kamm lab found that stresses due to IFs around the cell could activate integrins, which subsequently modulated cell migration within a 3D extracellular matrix^14,15^. Early work from our lab using a 3D microfluidic platform also indicated that IFs impacted tumor cell migration via an integrin mediated cell-ECM interaction^16^. Work from Tarbell’s lab demonstrated that IFs promoted tumor invasion via glycocalyx mediated mechanosensing^17^. More recently, the Munson lab showed that IFs can influence brain tumor cell invasion via both autologous chemotaxis (CXCR4/CXCL12 axis) and mechano-sensing (CD 44)^18^.

Malignant solid tumors typically appear as invasive cell aggregates lacking a basement membrane. Reduction or loss of cell-cell adhesion is an important biomarker for diagnosing tumor malignancy, particularly in the case of epithelial to mesenchymal transition (EMT)^19,20^. To our knowledge, studies of the impact of IFs on tumor invasion have been largely limited to single cell assays where single tumor cells were embedded within collagen matrices, i.e. only cell-ECM adhesions were considered^12–17,21–24^. In this paper, we have developed a 3D microfluidic co-culture tumor spheroid assay where tumor spheroids were embedded within 3D collagen matrices, and investigated whether/how IFs can modulate cell-cell adhesion within tumor spheroids in the context of tumor invasion. We introduce a co-culture spheroid model that provides direct physical cell-cell contacts mediated by cell-cell adhesions as well as a close mimic to the *in vivo* early stage of avascular breast tumors^25^. The co-culture spheroid consists of a 1:1 mixture of human metastatic breast tumor (MDA-MB-231 cell line) and non-tumorigenic breast epithelial (MCF-10A cell line) cells. A microfluidic platform was adapted to provide well defined IFs around the tumor spheroids and through the three dimensional (3D) architectural support (type I collagen) within the tumor microenvironment. We recognize the importance of tumor pressure and hydrodynamic flow within the tumor in tumor invasion^26,27^. Here we note that our work focuses on the roles of IFs within the stroma and around the avascular spheroid on tumor invasion. We find that IFs can significantly downregulate the cell-cell adhesion of non-tumorigenic cells in a co-culture spheroid and subsequently promote spheroid dissociation and invasion within a 3D ECM.

## Results

### Interstitial flows promote co-culture tumor spheroid dissociation

**Figure 1:**
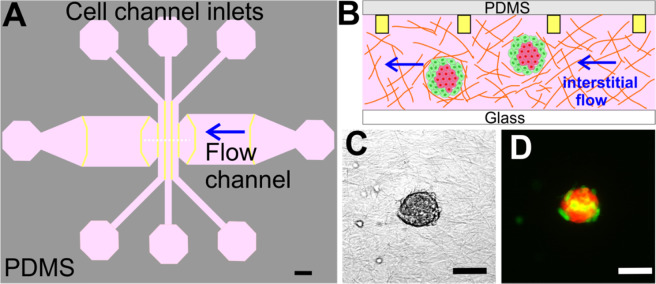
Microfluidic platform for tumor spheroid invasion. (**A**) Top view of the microfluidic device design with three cell channels and a flow channel. Spheroid embedded collagen matrices were introduced into the three cell channels and the flow channel and interstitial flows were introduced through the flow channel as indicated by the blue arrow. Yellow lines mark the contact lines. Each cell channel (distance between two straight yellow lines) is 400 µm wide and the flow channel is 3.0 mm wide, with 200 µm in depth and the contact line is 10 µm ×5 µm in cross section. Scale bar is 1 *mm*. (**B**). Cross sectional diagram (indicated by the white dashed line in A) of the microfluidic device showing spheroid embedded collagen within the cell channels. Yellow rectangles are cross sections of the contact lines and are not to scale. (**C** and **D**). Micrographs of a co-culture tumor spheroid [1:1 ratio of MDA-MB-231 (green) and MCF-10A (red) cells] embedded in type I collagen at a concentration of 1.5 mg/mL in bright field (**C**) and in fluorescence mode (**D**). Scale bar is 100 µm. CorelDRAW software was used to draw Fig. 1A,B.

**Figure 2:**
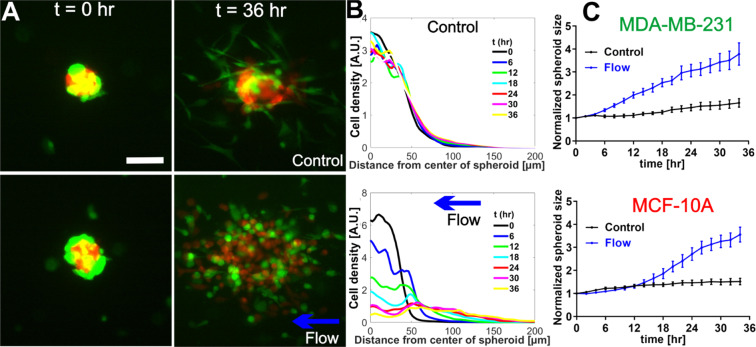
Interstitial flows promoted co-culture tumor spheroid dissociation. (**A**) Micrographs of co-culture tumor spheroids embedded in collagen matrices of 1.5 mg/mL at t = 0 hour (left panel) and t = 36 hours (right panel) in the absence (top panel) and presence (bottom panel) of the flow. Green: MDA-MB-231 cells expressing EGFP. Red: MCF-10A cells expressing dTomato variants. Scale bar is 100 µm. (**B**) Quantification of MCF-10A cell distributions using radial cell density in control (top) and flow (bottom). Here, cell density along the y-axis is computed using the azimuthal average fluorescence intensity at a specific radial distance from the center of the spheroid. Each colored line is a radial cell density profile at a specific time point, with t = 0 about 2 hours after the spheroids were introduced into the collagen matrices (**C**) Normalized spheroid size time evolution for MDA-MB-231 (top) and MCF-10A cells (bottom) with and without flow. Spheroid size is determined by fitting the radial cell density profile to a Gaussian function, and the sigma value is extracted as the spheroid size. Normalized spheroid size is computed as the spheroid size divided by the initial spheroid size at t = 0. We note the initial MDA-MB-231 spheroid size is about 20 percent larger than those of MCF-10A. Interstitial flows significantly increased spheroid size to almost two-fold of its original size. N = 17 spheroids (no flow) and N = 11 spheroids (flow) were used to generate the statistics. Mean and standard error of the mean are shown. ImageJ was used to prepare Fig. 2A.

To recreate the complexity of the tumor microenvironment, we embedded co-culture spheroids within a type I collagen gel using a flow based microfluidics system developed earlier in our lab^28^ (Fig. 1A,B). The co-culture spheroids consisted of malignant breast tumor cells (MDA-MB-231 cell line) and non-tumorigenic epithelial cells (MCF-10A cell line) (Fig. 1C,D), representing the cell diversity within the tumor microenvironment^29,30^. More importantly, the spheroid model provided physical cell-cell contacts mediated by cell-cell adhesions typically present in the *in vivo* environment, in contrast to the previous 3D microfluidic tumor cell models where single cells were embedded within an ECM^16^. We applied interstitial flow around the spheroids at a flow speed of 2.0 µm/s to mimic the interstitial flow within the tumor microenvironment. The flow direction is perpendicular to the cell channel (See arrow in Fig. 1A), and there is no flow in other directions. When observing co-culture spheroids within type I collagen gel in the presence of IFs, a striking phenomenon was immediately evident in that both cell types in the co-culture spheroids dissociated in the presence of IFs in contrast to the no flow case (control) during the 36 hour imaging time window (See Fig. 2, Fig. S1, and Movies S1 and S2). In the case of no flow (control, top panels of Fig. 2A), the majority of the MCF-10A cells stayed within the spheroid core and the peripheral MDA-MB-231 tumor cells invaded outwards. In the case of flow (lower panels of Fig. 2A), both MDA-MB-231 cells (Green) and MCF-10A cells (Red) spread out leaving no spheroid core behind.

To quantify the co-culture tumor spheroid dissociation, we computed the radial cell density profile using the azimuthal average of the cell fluorescence with respect to the center of the spheroid, the (0,0) coordinate. The time evolution of the radial cell density profiles for MCF-10A cells is shown in Fig. 2B in control (top) and flow (bottom). In control, the radial cell density profiles at different time points were about the same, which was consistent with Fig. 2A (top panel) where MCF-10A cells remained intact within the spheroid core. In the presence of the flow, the radial cell density profile spread out distinctly as time progressed, indicating that the MCF-10A cells invaded away from the spheroid center and the spheroid core disappeared towards the end of the experiment. The radial cell density profiles for MDA-MB-231 cells are shown in Fig. S2A.

To calculate the spheroid size, we fitted the radial cell density profile shown in Fig. 2B to a Gaussian function (see Fig. S2C). The fitted sigma value of the Gaussian function was used as the spheroid radius (or size) as shown in Fig. 2C. We note that tumor spheroid size (radius) here includes the spheroids before and after dissociation and represents a region in which 2/3 of the cell population resides. In Fig. 2C, spheroid size for both MDA-MB-231 cells and MCF-10A cells increased in the presence of flow in contrast to the no flow case. For MDA-MB-231 cells, the normalized average spheroid size was about 2-fold larger in flow (3.8 $\pm$0.49 fold) compared to no flow (1.7 $\pm$ 0.18 fold) at t = 36 hours (Fig. 2C top panel). For MCF-10A cells, the average normalized spheroid size was also about 2-fold larger in flow (3.6 $\pm$ 0.32 fold) compared to no flow (1.5 $\pm$ 0.14 fold) at t = 36 hours (Fig. 2C bottom panel).

Our results show that IFs increased the normalized spheroid sizes almost 2 fold compared to the control, no flow case for both cell types. We note that the spheroid dissociation had about 14 hours delay in the case of MCF-10A cells in contrast to about 4 hours delay for MDA-MB-231 cells (Fig. 2C). This pronounced difference indicated that the adhesion forces for keeping the MDA-MB-231 cells or MCF-10A cells within the spheroid core were different. We conjectured that the dominant force that kept the spheroid together was cell-cell adhesion via E-cadherin of MCF-10A cells. We thus investigated further on whether/how IFs affected E-cadherin mediated cell-cell adhesion.

### Interstitial flows promote co-culture tumor spheroid dissociation via downregulating E-cadherin of MCF-10A cells

**Figure 3:**
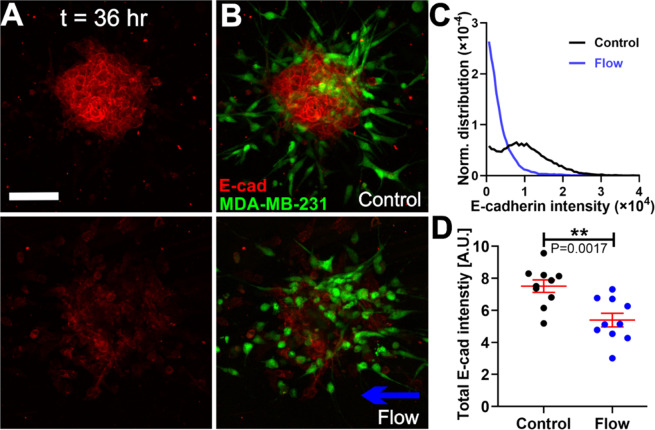
Interstitial flows promoted co-culture tumor spheroid dissociation via downregulating E-cadherin in MCF-10A cells (**A**) Micrographs of E-cadherin expression of co-culture spheroids using immunostaining in the absence (top) and presence (bottom) of flow. (**B**). Corresponding images of MDA-MB-231 cells expressing EGFP (Green) superimposed onto the E-cadherin expression image of the co-culture spheroid shown in the absence (top) and presence of flow (bottom). Scale bar is 100 µm for A and B. (**C**) Normalized distributions of E-cadherin intensity of a co-culture spheroid in control and flow. (**D**) Total E-cadherin intensity of the co-culture spheroids in control and flow condition. N = 10 spheroids in each condition were measured. ImageJ was used to prepare Fig. 3A,B.

E-cadherin expression of MCF-10A cells and MDA-MB-231 cells was measured using a standard immunostaining method. Figure 3A shows the E-cadherin expression of the co-culture spheroids after 36 hours of invasion in the absence (top panel) and presence of flow (low panel), in which the images are the maximum projection of the E-cadherin intensity from a z-stack of 5 to 6 images with a slice thickness of 13.36 µm (see Fig. S3 for single image slices from a sample z-stack). To examine the relationship between E-cadherin expression and the cell types, we superimposed an image of green fluorescence of MDA-MB-231 EGFP cells onto the image of red E-cadherin fluorescence of the co-culture spheroid (See Fig. 3B). Figure 3B (Also Fig. S4) shows that E-cadherin is mostly expressed on MCF-10A cells, with almost none on MDA-MB-231 cells, consistent with the reports from current literature^31,32^. We then quantified the E-cadherin intensity of the co-culture spheroids in control and flow conditions using the z-stack of fluorescent confocal images (See Fig. S3). We first computed the total E-cadherin fluorescence from all the image slices in one z-stack and plotted the normalized E-cadherin intensity distributions (See Fig. 3C). Figure 3C shows that IFs shifted the distribution to lower E-cadherin intensity values in comparison to the control case of no flow, with total E-cadherin expression significantly lower in flow (Fig. 3D). In addition, we observed that dissociated single MCF-10A cells expressed much lower E-cadherin expression compared to those residing within the spheroid or cluster, indicating MCF-10A cells may have lost E-cadherin when dissociating from the spheroid. Evaluating the E-cadherin distribution within single MCF-10A cells, we found that interstitial flows can also stimulate redistribution of the Ecadherin spatially within the cell (Fig. S5). These data suggest that IFs downregulated cell-cell adhesion via reducing E-cadherin expression as well as altering the E-cadherin localization within the MCF-10A cells, and as a result, the MCF-10A cells disassociated from the spheroid core in the presence of the flow.

### Interstitial flows modulate the modes of invasion of metastatic MDA-MB-231 and non-tumorigenic MCF-10A cells

To further understand the roles of IFs in co-culture spheroid dissociation and invasion, we investigated cell shape and motility in the presence and absence of the flow. Interestingly, we found that metastatic MDA-MB-231 cells and non-tumorigenic MCF-10A cells responded to IFs differently.

#### Interstitial flows promote an amoeboid over mesenchymal cell motility of the malignant MDA-MB-231 cells while having minimal impact on MCF-10A cell morphology

**Figure 4:**
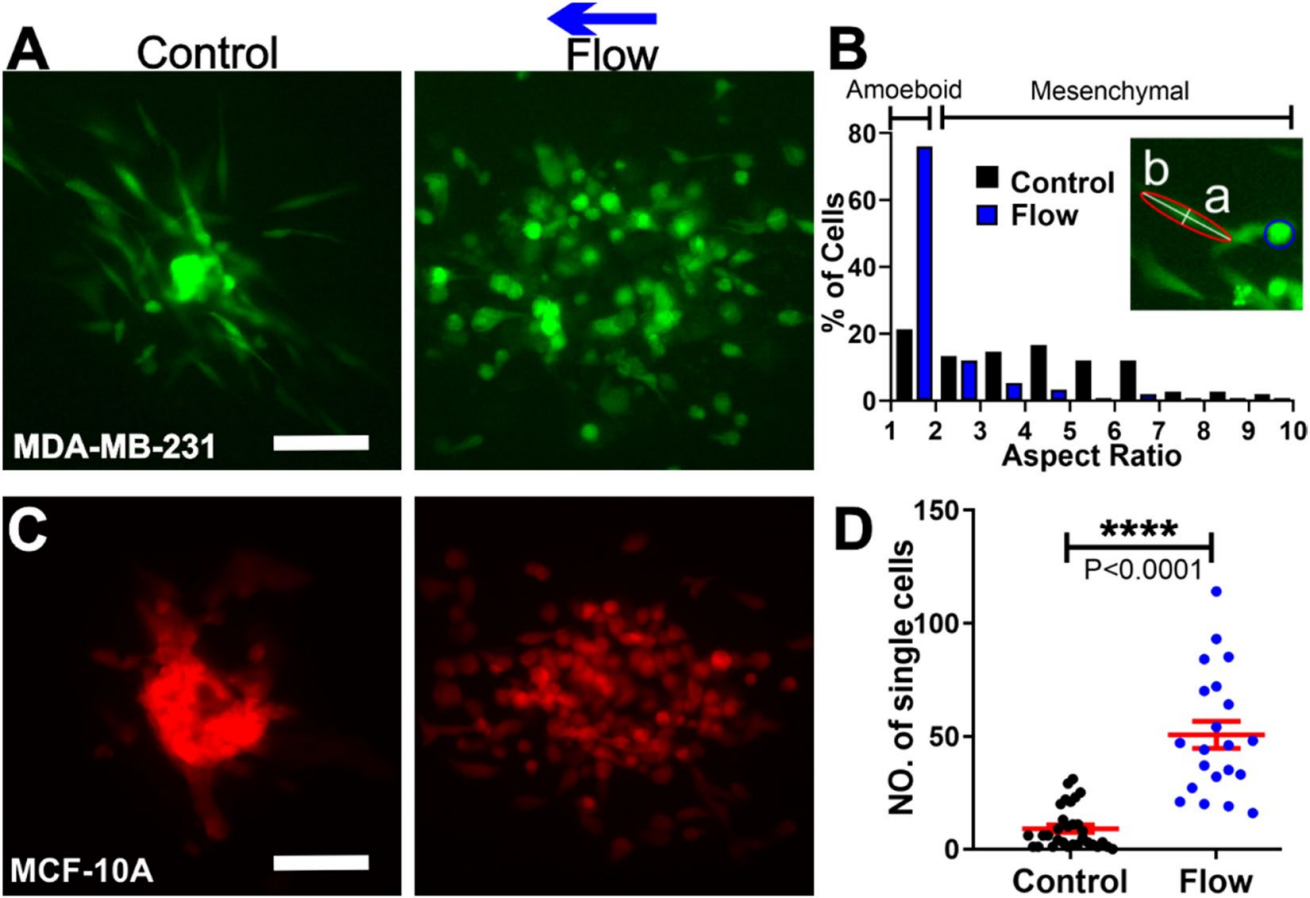
Interstitial flows modulated the modes of invasion of metastatic MDA-MB-231 and non-tumorigenic MCF-10A cells. (**A**) Micrographs of MDA-MB-231 cells expressing EGFP invaded out of the co-culture spheroids at t = 36 hr in the absence (left) and presence (right) of flow. Scale bar is 100 µm. (**B**) Aspect ratio distribution for MDA-MB-231 cells in the absence/presence of flow. The insert image illustrates the definition of aspect ratio, the ratio of major over minor axis of the cell (b/a). 150 cells for each condition per experiment were analyzed. (**C**) Micrographs of MCF-10A cells expressing dTomato in co-culture spheroids at t = 36 hr in the absence (left) and presence (right) of flow. Scale bar is 100 µm. (**D**) Number of single MCF-10A cells dissociated from the co-culture spheroids in the absence/presence of the flow. N = 3 to 5 spheroids were analyzed in each condition. ImageJ was used to prepare Fig. 4A,C.

In the presence of flow, MDA-MB-231 cells were found to be more round in shape and executed amoeboid motility in contrast to the case with no flow where cells were more elongated and mesenchymal like (Fig. 4A, Fig. S1 and S4, movies S3 and S4). We found that IFs shifted the aspect ratio of the cells towards smaller values as shown in Fig. 4B. Furthermore, IFs decreased the percentage of mesenchymal cells from 79 $\pm \,5\,$% in control to 24 $\pm \,5\,$% in flow. Here, amoeboid cells are defined as cells with aspect ratios smaller than 2.0 as suggested in the current literature^16^. This result is consistent with our early work where a 3D microfluidic single cell assay was used^16^. In contrast, MCF-10A cell morphology did not change significantly in the presence and absence of the flow (See Fig. S6).

#### Interstitial flows promote a single over collective cell motility of non-tumorigenic MCF-10A cells

In the absence of flow, majority of the MCF-10A cells stayed connected within the spheroid core. Cells were seen actively moving in a random way, always in direct contact with one another, consistent with collective migration as defined previously^33^ (See Fig. 4C left image and Movie S5). In the presence of flow, MCF-10A cells disassociated from the spheroid core and migrated as single cells (Fig. 4C right image and Movie S6). The total number of single MCF-10A cells disassociated from each co-culture spheroid was significantly higher in the flow than in the control case (See Fig. 4D). This result is consistent with our observation that IFs downregulated the E-cadherin of the MCF-10A cells as shown in Fig. 3.

### Interstitial flows significantly enhanced the motility of both the metastatic MDA-MB-231 and non-tumorigenic MCF-10A cells

**Figure 5:**
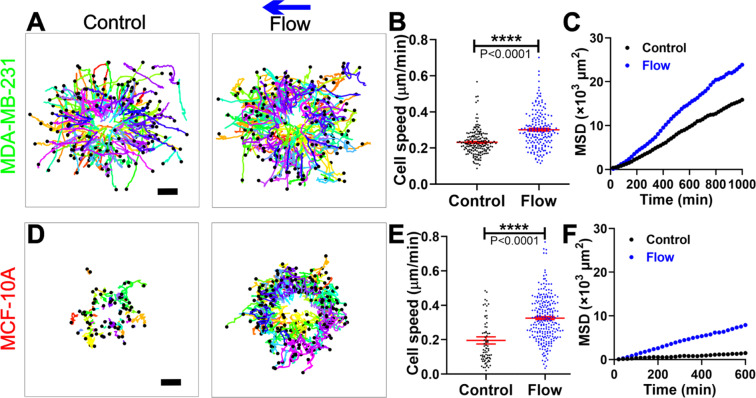
Interstitial flows significantly enhanced the motility of both the metastatic MDA-MB-231 and non-tumorigenic MCF-10A cells. (**A**) Trajectories of MDA-MB-231 cells invading out of the co-culture tumor spheroid in control (left) and flow (right). 180 cell trajectories were presented in each condition. The time duration of the trajectories is 11–35 hours (average of 22 hours) for control and 8–35 hours (average of 27 hours) for flow case. Scale bar is 100 µm. (**B**) Tumor cell migration speed for MDA-MB-231 cells with or without flow. (**C**) The mean squared displacements (MSDs) for MDA-MB-231 cells in control and flow. 169 cells in control and 121 cells in flow with time duration of 17 hours were used for the MSDs plot. (**D**) Trajectories of MCF-10A cells invading out of the co-culture tumor spheroid in control (left) and flow(right). The time duration of the trajectories is 2–31 hours (average of 14 hours) for control and 2–27 hours (average of 10 hours) for flow case. Scale bar is 100 µm. (**E**) Cell migration speed for MCF-10A cells with or without flow. (**F**). The MSDs for MCF-10A cells in control and flow. 46 cells in control and 107 cells in flow with time duration of 10 hours were used for the MSDs plot. Data in Fig. 5 are presented by combining three separate experiments. N = 3 to 5 spheroids were analyzed in each condition. Matlab was used to plot all the cell trajectories in A and D.

To quantify the MDA-MB-231 tumor cell motility from the co-culture spheroid experiments, we tracked each individual MDA-MB-231 cell that invaded out of the spheroids. Using the cell trajectories (Fig. 5A), we computed the tumor cell migration speed as well as the cell mean square displacements (MSDs). The cell migration speed was significantly enhanced by the flow, with an average speed of 0.30 $\pm$ 0.01 µm/min in flow in contrast to 0.23 $\pm$ 0.01 µm/min in control (Fig. 5B), or a 30% increase. To examine how far the tumor cells spread in space, we computed MSDs of the tumor cells and found that the MSDs were greater in the presence of flow than in the absence of the flow (Fig. 5C), indicating MDA-MB-231 tumor cells spread further in the presence of flow. Using the first order approximation for MSD where MSDs = 4D t^34^, we have the diffusion coefficient D = 6.41 $\pm$ 0.06 μm^2^/min for the flow case in contrast to 4.23 $\pm$ 0.03 μm^2^/min for no flow case. Note that we used the equation for a 2D system to estimate the diffusion coefficient because our cells were tracked in 2D although the cells were embedded within a 3D matrix. We also compared the cell migration velocity (Vx) and the persistence (Px) with respect to the flow direction in control and flow, but did not find any significant bias in the flow direction (Fig. S7). This result was similar to the results from our previous findings when single MDA-MB-231 tumor cells were embedded within type I collagen^16^.

Similarly, we analyzed the motility of the non-tumorigenic MCF-10A cells that were detached from the spheroid core and migrated individually out of the co-culture spheroids. We found that IFs also increased the speed and MSDs of the single MCF-10A cells, as shown in Fig. 5D–F. IFs increased the cell migration speed of MCF-10A cells from 0.20 ± 0.02 µm/min in control to 0.33 $\pm \,0.01$ µm/min in the flow case (Fig. 5E). The diffusion coefficient of MCF-10A was measured to be 0.55 $\pm$ 0.02 μm^2^/min in control in contrast to 3.44 $\pm$ 0.04 μm^2^/min in flow. The number of single cells analyzed and presented in Fig. 4D was less in control than in the flow case because MCF-10A cells preferred to migrate collectively rather than individually in control. We note that the trajectories as well as the MSD of MCF-10A cells were much shorter than those MDA-MB-231 cells because MCF-10A cells took longer to dissociate from the spheroid than MDA-MB-231 cells prior to their migration within the ECM. This phenomenon was also reflected in tumor spheroid size change of the two cell types as shown in Fig. 2C.

## Discussion

In this paper, we developed a 3D microfluidic tumor spheroid model for quantitative studies of the roles of IFs in tumor invasion. The microfluidic model represents an advance in *in vitro* tumor invasion models in that the cell-cell interactions within the tumor were considered in the presence of IFs. Our results demonstrated that IFs promoted tumor invasion through downregulating E-cadherin of the neighboring non-tumorigenic cells, highlighting the importance of external biophysical forces and a co-culture setting. To date, the roles of mechanical cues within the tumor microenvironment in tumor progression, aside from integrin mediated cell-ECM interactions^35,36^, are poorly understood. Our work emphasizes the importance of E-cadherin mediated cell-cell adhesion in tumor invasion, which is in agreement with recent publications on the importance of the interplay between cell traction force^37^, cell-cell^38,39^, and cell-ECM adhesions in regulating cell function. While our work focused on the roles of E-cadherin in the dissociation of tumor spheroids, recent work from the Ewald lab revealed that E-cadherin was required for the colonization of tumors at a foreign site^40^. We think our results are consistent with the results of the Ewald lab, since the downregulation we observe could function transiently to enable local invasion and then be restored at the metastatic site to enable metastatic growth. In future work, we will evaluate E-cadherin positive breast cancer cell lines to see if IFs induce reduction in them as well. These insights are important in future designs of *in vitro* as well as *in vivo* assays for cancer biology research, cancer diagnostics, and therapeutic drug testing.

Cell-cell interactions between MDA-MB-231 and MCF-10A cells are important in mediating tumor invasion. The impact of IFs on a pure MCF-10A spheroid is less pronounced than on co-culture spheroid invasion (See. Fig. S8A,C). In addition, the E-cadherin expression of MCF-10A using MCF-10A alone spheroids did not show a significant difference between the presence or absence of flow (See Fig. S8B,D). This indicates that the MDA-MB-231 cells are required for the down-regulation of E-cadherin of MCF-10A cells in the presence of IFs. We speculate that the integrins on MDA-MB-231 cells that were in contact with the ECM were stimulated by IFs as reported in prior work^14^, leading to secretion of factors (e.g. possibly TGF-beta), that subsequently triggered downregulation of E-cadherin in the neighboring MCF-10A cells. We note that pure MDA-MB-231 cells do not form spheroids under the same cell culture conditions. Although it is likely that MCF-10A cells can communicate with MDA-MB-231 cells chemically and physically in the presence of the flow, which subsequently promote MDA-MB-231 cell invasion, further investigations will be needed to fully understand the molecular interactions between MCF-10A and MDA-MB-231 cells in the presence of IFs in tumor invasion.

Looking forward, an important question to answer is whether/how IFs can regulate E-cadherin or cadherin-beta-catenin complex directly via mechano-sensing. To address this question, it will be important to explore a range of flow rates to determine whether a threshold for triggering invasion exists. Recent work showed that E-cadherin-beta-catenin complex can be regulated directly by mechanical stress^41,42^, with a 6pN force being sufficient to stimulate the E-cadherin-beta-catenin complex^43^. In our work, a flow rate of 2 µm/s creates about 0.04 pN shear force on an MCF-10A cell. We anticipate a much larger flow rate will be needed to stimulate the E-cadherin-beta-catenin complex. Related to this is whether physiological or only pathological flows impact tumor invasion. To address this question, a parallel advance needs to be made in measuring the physiological and pathological IF rates *in vivo*. Currently, only one measurement has been carried out in the rabbit ear model to show that physiological IFs ranged from 0–2.0 µm/s^4^. In contrast, a number of measurements have been reported for the IF rates in both human and animal tumors ranging from 0.1–55 µm/s^5,7,44^.

## Methods

### Cells, spheroids and 3D spheroid culture preparation

#### Cells

Metastatic breast adenocarcinoma cells (MDA-MB-231 cell line) and non-tumorigenic mammary epithelial cells (MCF-10A cell line) were provided by the Cornell Center of Microenvironment and Metastasis. MDA-MB-231 cells were cultured every 3 to 4 days from passage 2 to 20, and used at 50–70% confluency^45^. The growth medium for MDA-MB-231 cells was composed of DMEM high glucose medium (Catalog No. [Cat.] 11965092, Gibco, Life Technologies Corporation, Grand Island, NY), 10% fetal bovine serum (Cat. S11150, Atlanta biologicals, Lawenceville, GA), and 1% antibiotics (100 units/mL penicillin and 100 µg/mL streptomycin, Cat. 15140122, Gibco). MCF-10A cells were cultured every 3 to 4 days from passage 2 to 10, and used at 70–90% confluency. The growth medium for MCF-10A cells was composed of DMEM/F-12 medium (Cat. 11320033, Gibco), 5% donor horse serum (Cat. S12150, Atlanta biologicals), 20 ng/mL human EGF (Cat. PHG0311, Gibco), 0.5 µg/mL hydrocortisone (Cat. H0888–1G, Sigma-Aldrich, St. Louis, MO), 100 ng/mL Cholera Toxin (resuspend at 1 mg/ml in sterile DI ${{\rm{H}}}_{2}$O, Cat. C8052-.5MG, Sigma-Aldrich), 10 µg/mL insulin (Cat. 10516-5 ML, Sigma-Aldrich), and 5% antibiotics (Gibco). MDA-MB-231 expressing EGFP and MCF-10A cells expressing dTomato variants were kind gifts from Dr. Joseph Aslan at the Oregon Health & Science University. Fluorescently labeled MDA-MB-231 and MCF-10A cells were cultured in the same way as the non-labeled cells and were used for the co-culture spheroid experiments.

#### Spheroids

Tumor spheroids with uniform size were generated using a microfabricated microwell array platform (See Fig. S9 and also work from the Ma lab^46^). Briefly, each spheroid was formed within a 200 µm diameter and 220 µm height non-adherent microwell treated with 1% pluronic F-127 solution (Cat. P2443-250G, Sigma-Aldrich) (see Fig. S9A). A 36 by 36 microwell array was patterned in a thin PDMS membrane with a dimension of 1 cm $\times$ 1c m (Fig. S9B), and 6 of the microwell arrays were placed in 6 wells out of a 12-well plate (Fig. S9C). Within each well of the 12-well plate, 2 million cells (1:1 ratio of MDA-MB-231:MCF-10A) suspended in 2.5 mL medium (1:1 ratio of DMEM and DMEM/F12 growth media) were introduced. Cells were first allowed to settle down into all microwells for 30 minutes in the incubator before the device was placed on a rocker (Boekel Scientific, Rocker II Model 260350). Co-cultured spheroids were formed after overnight (see Fig S9A and movie S7) and cultured for 5 to 6 days before experiments, with medium change every 2–3 days. We note that the architecture of the co-culture spheroids evolves with time, in particular within the first few days^47^. It sensitively depends on cell adhesion, growth, and duration of the culture. The architecture with the MCF-10A in the core and MDA-MB-231in the periphery is likely formed to minimize the free energy associated with the differential adhesion among the MCF-10A and MDA-MB-231 cells^46,48^. Here, we systematically used the same cell culture condition, harvested spheroids at 5–6 days and selected spheroids of similar architecture (MCF-10A core, MDA-MB-231 cells outside) for data analysis. This design allowed us to generate about 1296 spheroids of approximately 100 µm in diameter in a robust way. We targeted this spheroid size so that spheroids can fit within the microfluidic device with the device height constraint of 200 µm.

#### Spheroid embedded ECM

To prepare spheroid embedded collagen matrices, 60 µL Type I collagen from stock concentration of 5.0 mg/mL (Cat. 354249, Corning, Discovery Labware Inc., Bedford, MA) was first titrated with 1.32 µL 1 N NaOH and 20 µL 10X M199 (Cat. M0650-100ML, Sigma-Aldrich) to yield a final pH of approximately 7.4^49^. The collagen was then mixed with the collected co-culture spheroids in 1:1 ratio medium (DMEM: DMEM/F12 growth medium) to a final volume of 200 µL. The final average spheroid concentration was approximately 33 spheroids per device (about 1 spheroid per $m{m}^{2}$ under the top view) and the final collagen concentration was 1.5 mg/mL. For MCF-10A spheroid preparation, the steps were the same as for the co-culture spheroids. Both DMEM/F12 growth medium and 1:1 ratio of DMEM:DMEM/F12 medium have been used for the MCF-10A spheroid invasion experiments. We note that tumor spheroids were collected from four arrays of microwells for each experiment and filtered by a Falcon Cell Strainer (Cat. 352350, Corning) with 70 µm pores to ensure the uniformity of the spheroid size.

#### Immunostaining of E-cadherin

MDA-MB-231 cells expressing EGFP and unlabeled MCF-10A cells were used to form co-culture spheroids and used in the microfluidics for both static and flow conditions. Immunostaining was performed within the microfluidics after the spheroid embedded collagen was subjected to the flow for 36 hours. Spheroids after invasion with/without flow were first fixed with 4% (v/v) paraformaldehyde (Cat. Sc-281692, Santa Cruz, Dallas, TX) in PBS (Cat. 10010023, Gibco) and washed three times with 1X wash buffer (0.05% Tween-20 in PBS, Cat. P9416-50ML, Sigma-Aldrich). The spheroids were then permeabilized with 1% (v/v) Triton X-100 (Cat. T8787-50ML, Sigma-Aldrich) in PBS, washed twice with 1X wash buffer, and blocked with 0.5% (v/v) Tween-20 with 3% BSA (Cat. 05470-1 G, Sigma-Aldrich) in PBS. Anti-E-cadherin primary antibody (Cat. ab1416, Abcam, Cambridge, MA) solution (1:50) in 1% (w/v) BSA in PBS was flowed through the spheroid embedded collagen and incubated for overnight at 4°C. After washing three times, Alexa Fluor 594 conjugated secondary antibody (Cat. ab150116, Abcam) (1:100) in 1% (w/v) BSA was flowed through the sample and incubated for 1.5 hours in room temperature. The samples were washed three times before imaging.

### Microfluidic design and experimental procedure

A microfluidic platform (See Fig. 1) previously developed in our lab was adapted to provide spatially uniform IFs around the spheroids embedded collagen matrices^28^. The original design of the device was to confine cell-matrices within the cell channels using the contact lines, where cells were confined in the cell channels only. In the spheroid experiments here, spheroid embedded collagen was introduced to both the cell and the flow channels to increase the experimental throughput (See Fig. 1A,B). IFs were introduced through the flow channel using a syringe pump (Kd Scientific, Model #:78-0230). There were three identical devices as shown in Fig. 1A patterned on a ($1\mbox{''}\,\times \,3\mbox{''}$ chip) for parallel experiments.

The silicon master of the microfluidic device was fabricated in the Cornell Nanofabrication Facility (CNF) using a two layer photo-lithography method. The PDMS device was made from the silicon master using a soft-lithography method. Details of the fabrication method can be seen in reference^28^. For sterility, PDMS devices were autoclaved and then treated with oxygen plasma (Harrick Plasma Cleaner PDC-001, Harrick Plasma, Ithaca, NY) for 1 minute on high power mode. To ensure optimal surface properties for binding with collagen, the PDMS devices along with a standard 1” × 3” size glass slides (Fisher Scientific, Pittsburgh, PA) were activated with 1% Poly(ethyleneimine) (Cat. P3143-100ML, Sigma-Aldrich) for 10 minutes followed by a 0.1% Glutaraldehyde (Cat. 16019, Electron Microscopy Sciences, Hatfield, PA) treatment for 30 minutes. The PDMS and glass slides were left in a biohood for overnight in room temperature after being rinsed three times. The PDMS device was then sandwiched between the glass slide and a plastic manifold. A 0.6% of agarose solution was used to fill the void space around the PDMS device to prevent medium from evaporation during spheroid invasion experiment. In a typical experiment, two such microfluidic chips were prepared in parallel and stored at 4 °C for 30 minutes for later use.

At the day of experiments, spheroid-embedded collagen solution was introduced to the cell channels and the flow channel in each of the three devices on a chip (See Fig. 1A,B) while the device was in direct contact with an ice block. The microfluidic device was then placed in a petri dish padded with wet tissues, and the petri dish was placed in an incubator to allow collagen polymerization for 45 minutes at 37 °C. We note that the temperature ramping rate during polymerization is a critical factor for collagen structure, faster warming leads to a uniform and small pore size network, while slow warming (adopted here) leads to an inhomogeneous large pore size network. More details can be seen in ref. ^50^). To prevent spheroids from gravitationally settling down to the bottom of the device, the microfluidic chips were positioned up-side-down for the first 10 minutes and then flipped three times more at time points of 5, 15, and 15 minutes in the incubator. Following polymerization, 37 °C 1:1 ratio medium was flowed into all the channels and both the inlets and outlets of the channels, then were plugged with PDMS filled gel loading tips. The microfluidic device was then transferred to the microscope stage enclosed by an environmental control chamber (WeatherStation, PrecisionControl LLC), which was kept at 37 °C, 5% CO2 and about 70% humidity. Right before imaging started, IFs (using 1:1 ratio of DMEM and DMEM/F12 growth media) were pumped through the flow channels for the flow cases. Here t = 0 is defined as when the flow was introduced, about 2 hours after the spheroid embedded collagen was polymerized. In each experiment, one device was used as control (no flow) and the other two were connected to a syringe pump to introduce flow of 0.05 µL/min (or 2.0 µm/s). Experiments were repeated three times.

### Imaging and data analysis

An inverted microscope (IX81, Olympus America, Center Valley, PA, USA) with a CCD camera (Orca-ER, Hamamatsu Photonics, Japan) was used for all the invasion experiments. In a typical experiment, the middle z-plane of the spheroids in the channels were captured using a 10X objective (Olympus, NA = 0.3) in bright field mode (Fig. 1C) and in green fluorescence mode (EX:460-500 nm, EM: 510–560 nm) for EGFP-MDA-MB-231 cells and red fluorescence (EX: 510–560 nm, EM: 572.5–647.5 nm) mode for dTomato-MCF-10A (Fig. 1D). A sequence of 109 images was captured every 20 minutes for a total of 36 hours.

To quantify tumor spheroid dissociation, fluorescent images of tumor spheroids was used to calculate spheroid sizes with an in house Matlab program^51^. Here, the radial cell density shown in Fig. S2A,B was computed using the azimuthal average fluorescent intensity of the cells at a specific radial distance from the center of the spheroid (Fig. S2A,B). Each cell density profile is for a specific time point, with t = 0 is about 2 hours after the spheroids were introduced into the collagen matrices. Each cell density profile was then fitted to a Gaussian function to obtain the sigma value to be used as the spheroid size or radius of the spheroid (Fig. S2C), which represents a region where 2/3 of the cells reside. All the spheroid sizes were then normalized to the initial spheroid size. We note that Gaussian function fitted best to our data.

To quantify E-cadherin expressed on the cells after invaded out of the spheroid, A Zeiss LSM 710 confocal microscope was used to image the immunostained E-cadherin at the end time point t = 36 hours. Typically, a z-stack of 5–6 slices fluorescence images (See Fig. S3) with each slice thickness of 13.36 µm was taken for each co-culture spheroid using a 10X objective (Pinhole setting is AU = 1.01, and EX:561 nm, EM:659 nm for immunostained E-cadherin, and EX:488 nm, EM:536 nm for EGFP-MDA-MB-231cells). This imaging modality is chosen to ensure that the z-stack captures all the E-cadherin fluorescent signals from the cells of the same initial spheroid, with a field of view of 425 µm × 425 µm × 100 µm. To quantify the E-cadherin intensity, the Z-stack images were projected onto a 2D image and all the pixel intensities were added up using the Z Project function in ImageJ. Using this method, each 2D image contained all the fluorescence emitted from the immunostained E-cadherin of the spheroid. After subtracting the background, normalized distributions of E-cadherin fluorescence for control and flow were plotted and the total E-cadherin fluorescence for 10 spheroids were compared in control and flow. We note that the total E-cadherin expression was slightly overestimated for both control and flow because of a 4.4 µm overlap between two consecutive z-slices.

To quantify tumor cell morphology, an ellipse function from ImageJ was used to fit a single cell. The aspect ratio was obtained as the major over minor axis of the fitted ellipse. Cells with aspect ratio greater or equal to 2 were considered mesenchymal cells, and otherwise amoeboid cells^16^.

To quantify tumor cell motility, time-lapse images of tumor spheroid invasion were processed in ImageJ and in house Matlab programs. MDA-MB-231 and MCF-10A cells were tracked after they invaded out of the spheroid. Note that the starting time for each cell differed because each cell invaded out from the spheroid at different time points. All the tracked trajectories were used to compute the cell migration speed and the mean square displacements (MSDs)^16^.

### Statistical analysis

All the data were plotted using Matlab or Prism GraphPad software. Student’s t-test was performed for two-group analysis using Prism and mean ± SEM were presented in all numerical results as well as the average line and error bars in the plots.

## Supplementary information

Supplementary Information.

Supplementary Movie S1

Supplementary Movie S2

Supplementary Movie S3

Supplementary Movie S4

Supplementary Movie S5

Supplementary Movie S6

Supplementary Movie S7

## Data Availability

All the raw data and analyzing tools for this study are available upon request to the corresponding author.
