# Supplementary material for: Tumor spheroids under perfusion within a 3D microfluidic platform reveal critical roles of cell-cell adhesion in tumor invasion
Source: Sci Rep. 2020 Jun 15;10:9648. doi: 10.1038/s41598-020-66528-2 (PMC7295764; doi:10.1038/s41598-020-66528-2)
Supplement: Supplementary file 1 — Supplementary Information. [file 41598_2020_66528_MOESM1_ESM.docx]

Tumor spheroids under perfusion within a 3D microfluidic platform revealed critical roles of cell-cell adhesion in tumor invasion

Yu Ling Huang ^1^, Yujie Ma^1†^, Cindy Wu ^1^, Carina Shiau ^1^, Jeffrey E. Segall ^2^, and Mingming Wu ^1*^


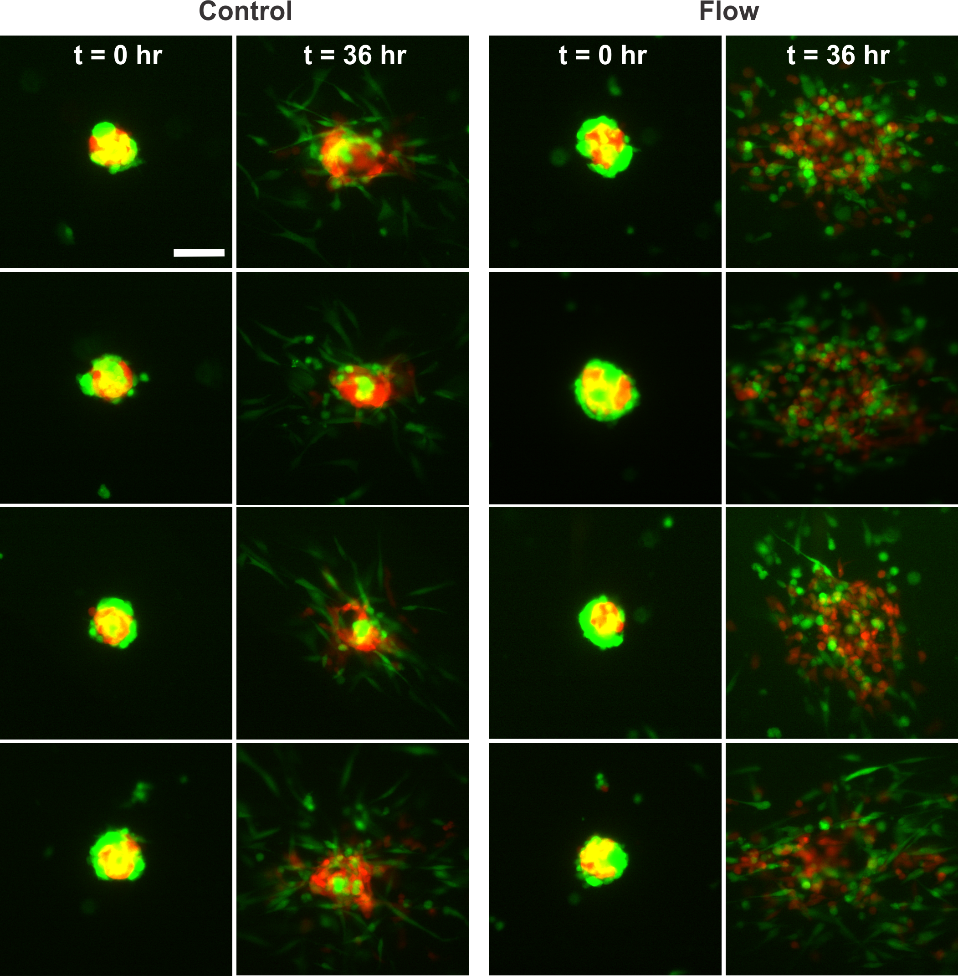


**Figure S1:** **Micrographs of co-culture tumor spheroid invasion in control and flow**. 4 individual spheroid invasion events at t = 0 and 36 hours are shown for both control and flow case. MDA-MB-231 cells are green expressing EGFP and MCF-10A cells are red expressing dTomato. Scale bar is 100 µm.


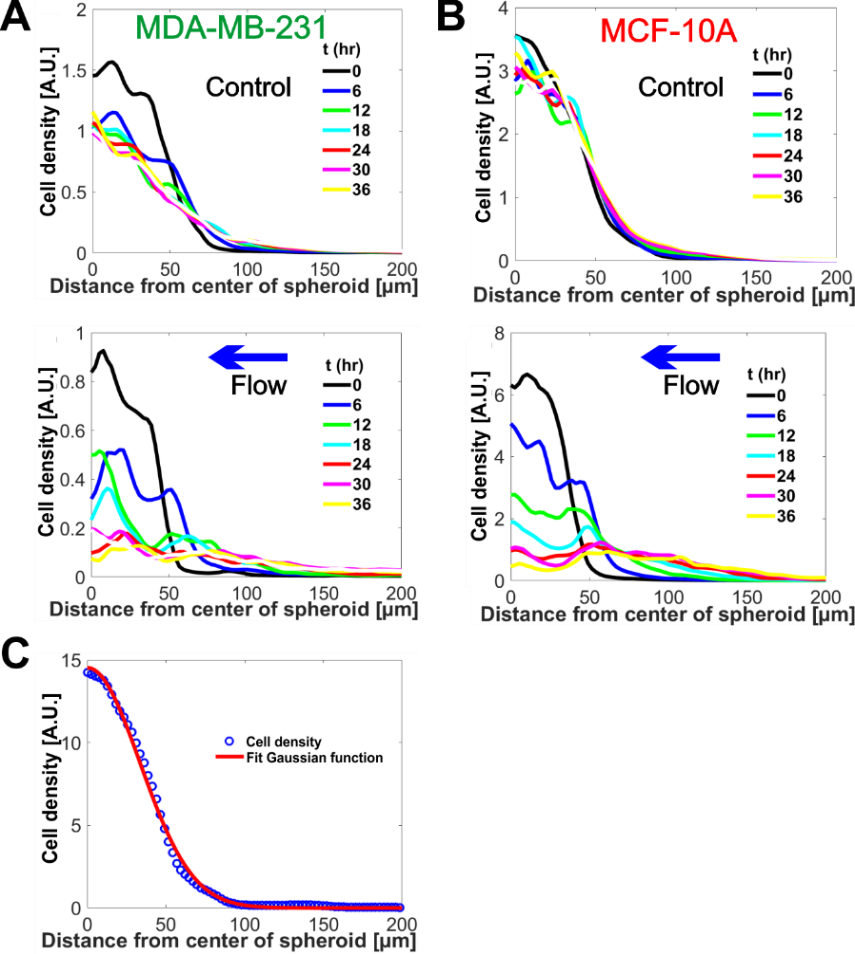


**Figure S2:** **Time evolution of radial cell density profile to quantify tumor spheroid dissociation** **using azimuthally averaged radial cell intensity.** A, B. The radial cell density at various time points for MDA-MB-231 cells (A) and MCF-10A cells (B) in control (top panel) and flow (bottom panel). C. A radial cell density profile (at t=0 for MCF-10A cells) is fitted to a Gaussian function. The fitted sigma value is used as the size (radius) of the spheroid, which represents the region where 2/3 of the cells reside.


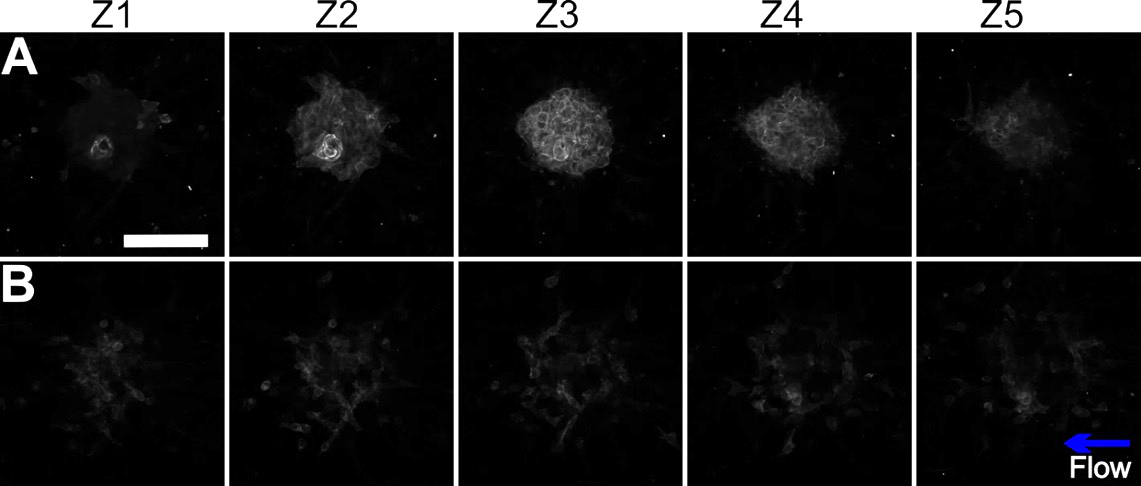


**Figure S3:** Confocal images of E-cadherin in single slices from a z-stack of the co-culture spheroids in control (A) and flow (B). Each image slice has a thickness of 13.36 µm.


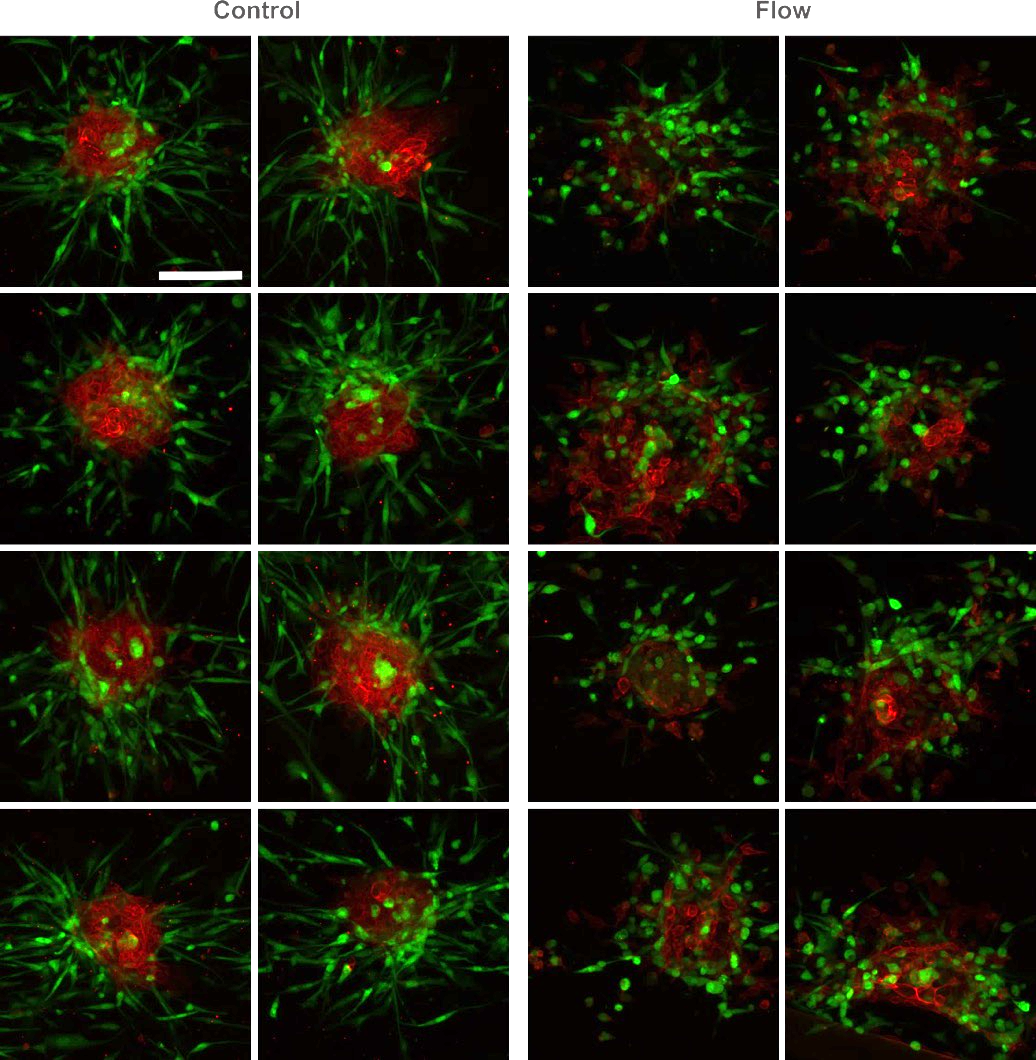


**Figure S4:** **Micrographs of superimposed images of immunostained Ecadherin in co-culture spheroids with MDA-MB-231 cells expressing EGFP of 8 invading spheroids under no flow or after 36 hours of flow.** Green: MDA-MB-231 cells. Red: immunostained Ecadherin.


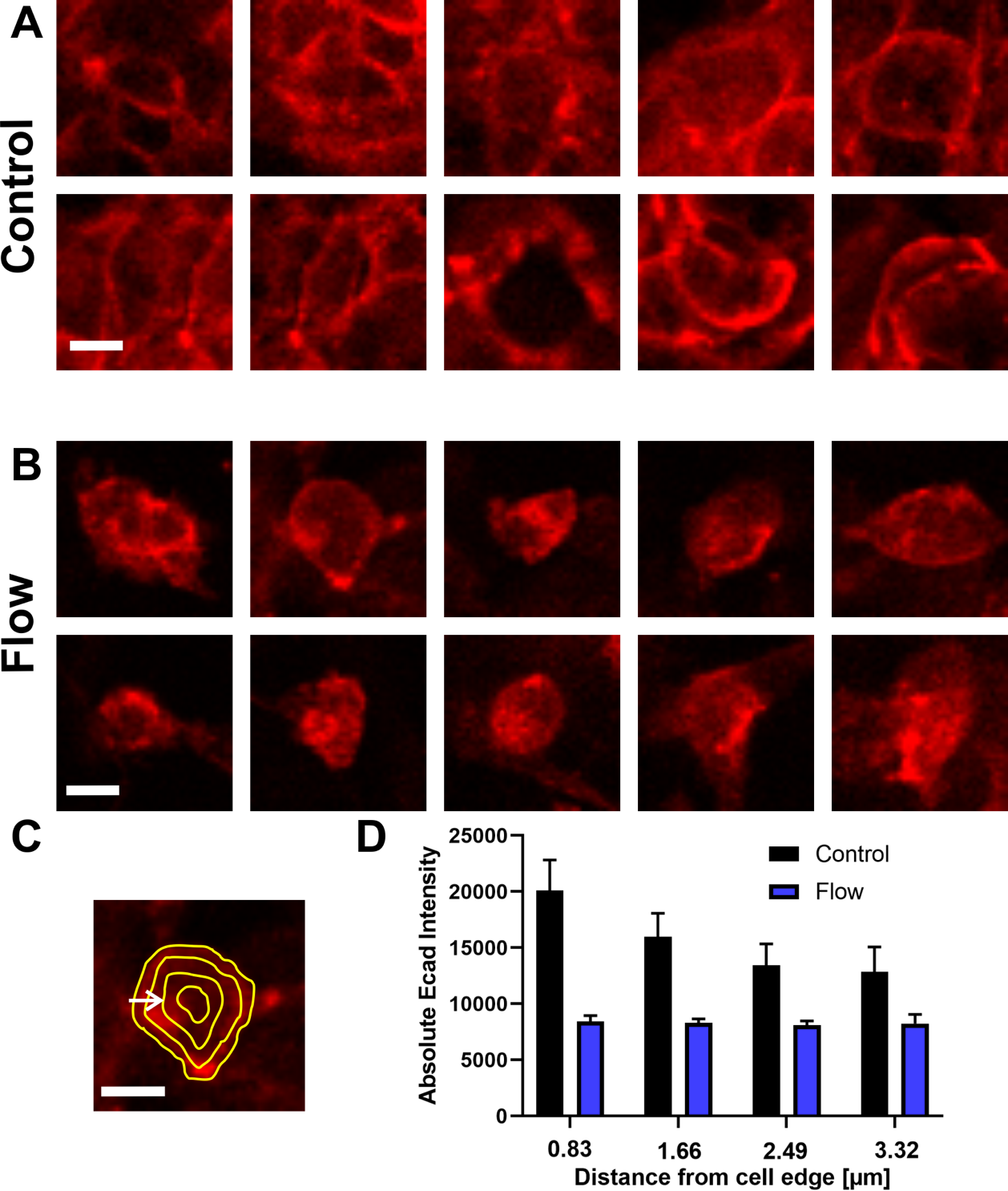


Figure S5: Interstitial flow regulated E-cadherin spatial distribution within single MCF-10A cells. A.B. E-cadherin immunostaining of MCF-10A cells within co-culture spheroids in control (A) and individual MCF-10A cells that have detached from the spheroid in the presence of flow (B). Images were taken by confocal microscopy (Zeiss LSM 710) with 10X objective. The images were from single z-slice, cropped and enhanced brightness to show single cell expression of E-cadherin. Scale bar is 10 µm. C. Method to measure the E-cadherin intensity distribution for a MCF-10A cell. Each cell was outlined from the edge (yellow outline) and the outline was reduced inwards (indicated by white arrow) in one pixel (or 0.83 µm) step towards the center. The average intensity between two outlines was measured and used to calculate the average intensity per pixel at that distance from the edge. Scale bar is 10 µm. D. Absolute E-cadherin intensity at step distances from the cell edge. 10 MCF-10A cells for each condition were used for the analysis using the raw unenhanced intensity data.


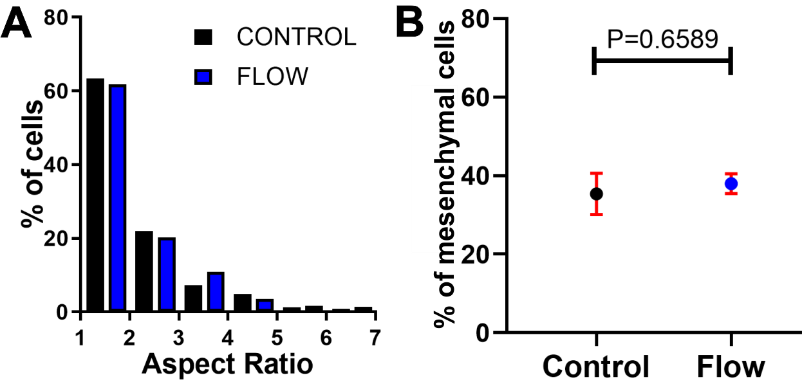


**Figure S6**: **Interstitial flows have no significant impact on single MCF-10A cell morphology for those invaded out of the co-culture spheroids.** Aspect ratio distribution (A) and percentage of mesenchymal cells (B) of MCF-10A cells in control and flow.


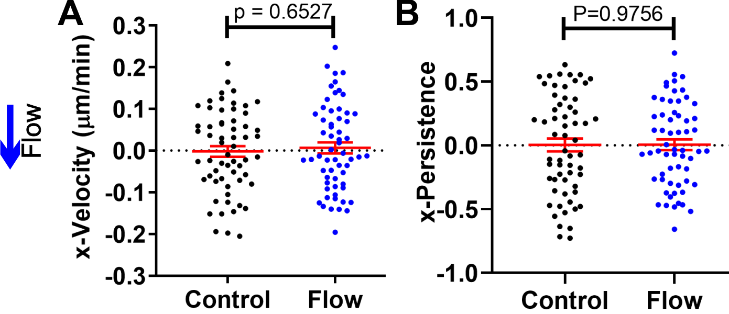


**Figure S7:** **No significant directional cell migration was observed for MDA-MB-231 tumor cells from the co-culture spheroids.** A. Tumor cell velocity in the flow direction. Control: Vx = -0.0019 $\pm$ 0.013 µm/min. Flow: Vx = 0.0065 $\pm$ 0.014 µm/min. B. Tumor cell persistence in the flow direction. Control: Px = 0.0035 $\pm$ 0.051. Flow: Px = 0.0055 $\pm$ 0.043. Positive sign indicates a direction against the flow and negative sign indicates a direction that is along the flow direction.


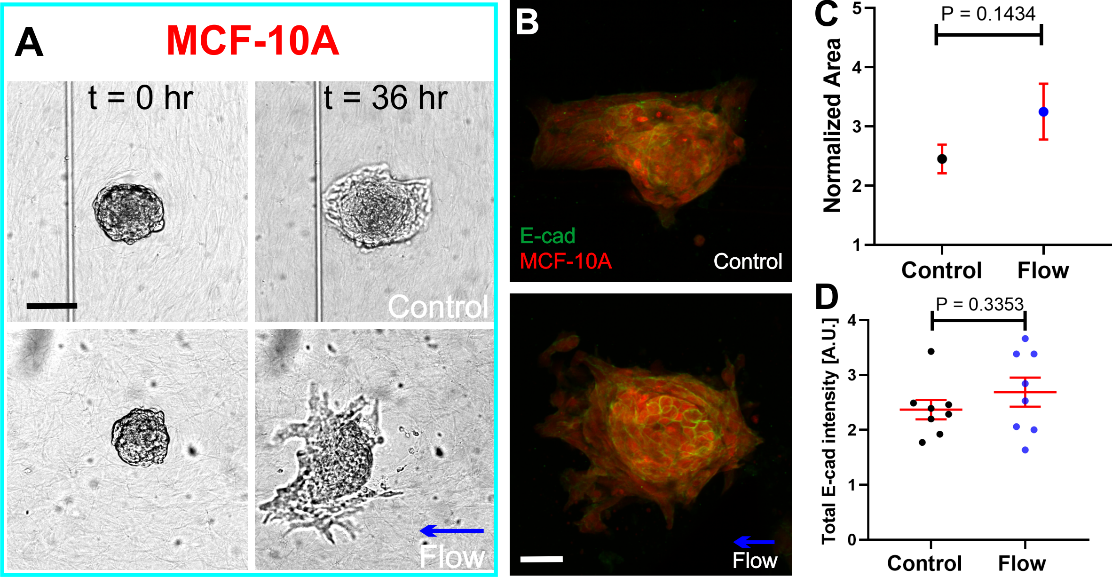


**Figure S8: Interstitial flow has no significant impact on E-cadherin expression in MCF-10A alone spheroid invasion.** A. Micrographs of MCF-10A spheroids embedded in collagen matrices of 1.5 mg/mL at t = 0 hour (left panel) and t = 36 hour (right panel) in the absence (top panel) and presence (bottom panel) of the flow. Scale bar is 100 µm. B. Confocal images of MCF-10A spheroids after 36 hours of invasion in the absence (top panel) and presence (bottom panel) of flow. Red: MCF-10A cells; Green: E-cadherin. Scale bar is 50 µm. C. Normalized area of the MCF-10A spheroids after invasion for control and flow at t = 36 hr. N = 14 spheroids were analyzed in each condition. D. Total E-cadherin intensity for MCF-10A spheroids at the end of the flow experiment at t = 36 hr. N = 8 spheroids were analyzed in each condition.


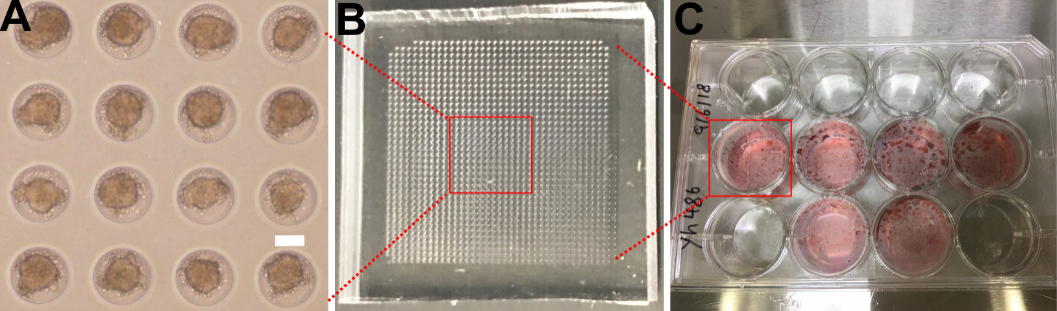


**Figure S9: Robust and high throughput tumor spheroid formation assay.** A. Micrograph of tumor spheroid formation within the microwells. Each microwell has a diameter of 200 µm and a depth of 220 µm. B. A PDMS device (1cm by 1cm) contains an array of 36 by 36 microwells, each microwell can culture one spheroid. C. 12-Well plate is used for spheroid formation and growth. 6 out of the 12 wells contain a PDMS spheroid formation device. Typically, spheroids collected from 4 PDMS arrays are used for each experiment. Scale bar is 100 µm. Fig. A was prepared using ImageJ.

**Movie Legends**

Movie S1: **Co-culture tumor spheroid invasion within 1.5mg/mL collagen in the absence of flow**. Each image is 420 µm $\times$420 µm, the time between consecutive image is 20 minutes, and the duration of the move is 36 hours. Green: MDA-MB-231 cells. Red: MCF-10A cells.

Movie S2: **Co-culture tumor spheroid invasion within 1.5mg/mL collagen in the presence of flow**. Each image is 420 µm $\times$420 µm, the time between consecutive image is 20 minutes, and the duration of the move is 36 hours. Green: MDA-MB-231 cells. Red: MCF-10A cells.

Movie S3: **MDA-MB-231 cell invasion from the co-culture tumor spheroid within 1.5mg/mL collagen in the absence of flow**. Each image is 420 µm $\times$420 µm, the time between consecutive image is 20 minutes, and the duration of the move is 36 hours.

Movie S4: **MDA-MB-231 cell invasion from the co-culture tumor spheroid within 1.5mg/mL collagen in the presence of flow**. Each image is 420 µm $\times$420 µm, the time between consecutive image is 20 minutes, and the duration of the move is 36 hours.

Movie S5: **MCF-10A collective cell invasion from the co-culture tumor spheroid within 1.5mg/mL collagen in the absence of flow**. Each image is 420 µm $\times$420 µm, the time between consecutive image is 20 minutes, and the duration of the move is 36 hours.

Movie S6: **MCF-10A cell invasion as single cells from the co-culture tumor spheroid within 1.5mg/mL collagen in the presence of flow**. Each image is 420 µm $\times$420 µm, the time between consecutive image is 20 minutes, and the duration of the move is 36 hours.

Movie S7: **Co-culture tumor spheroid formation within the microwells** (without rocking). Each image is 740 µm $\times$490 µm, the time between consecutive image is 15 minutes, and the duration of the move is 24 hours. Green: MDA-MB-231 cells. Red: MCF-10A cells.
